# 3-D assessment of skeletal and dentoalveolar bilateral dimensions in unilateral impacted palatal canine cases – A CBCT study

**DOI:** 10.4317/jced.60982

**Published:** 2023-12-01

**Authors:** Swati Sharma, Payal Sharma, Akshay Rathore, Monis Raza

**Affiliations:** 1 Assistant Professor, Department of Orthodontics & Dentofacial Orthopedics. Institute of Dental Studies and Technologies, Ghaziabad (U.P), India; 2 Professor & Head, Department of Orthodontics & Dentofacial Orthopedics. I.T.S. Center for Dental Studies and Research Muradnagar, Ghaziabad (U.P) - 201206, India; 3 Professor, Department of Oral Medicine & Radiology, I.T.S. Center for Dental Studies and Research Muradnagar, Ghaziabad (U.P) - 201206, India; 4 Associate Professor, Department of Orthodontics & Dentofacial Orthopedics. Institute of Dental Studies and Technologies, Ghaziabad (U.P), India

## Abstract

**Background:**

The aim of this study was to compare skeletal and dentoalveolar dimensions in subjects with maxillary unilateral impacted palatal canines versus the unaffected contralateral side using CBCT.

**Material and Methods:**

Skeletal and dentoalveolar variables (Anterior alveolar ridge height, Anterior dentoalveolar height , nasal cavity width, basal nasal width, Lateral angulation of long axis of the incisors and canines with respect to the nasal horizontal plane, premolar to median raphe width, dimensions of lateral incisor and canine, root resorption of lateral incisors, crown-root angulation of lateral incisor, and sector classification of canine) were compared between the impacted and the contralateral sides. As the data had normal distribution, means were compared using students t test. The significance was set at *p*<0.05. The root resorption in lateral incisor was compared using Chi square test.

**Results:**

Lateral angulation of long axis of canines, nasal cavity width, basal lateral width, and premolar to median raphe width were found to be significantly different. Maximum number fell in sector 4 (n = 23, 38.3%) in sector classification. Root resorption of lateral incisor on impacted side was insignificant.

**Conclusions:**

Skeletal and dento-alveolar dimensions vary between the impacted and non-impacted sides in unilateral palatal canine impaction cases. Canines on the impacted side were more mesially angulated compared to the non-impacted side. The nasal cavity width, basal lateral width and premolar to median raphe width were significantly less on the impacted side compared to the non- impacted side.

** Key words:**Impacted canine, CBCT, skeletal dimensions, diagnosis, orthodontic treatment.

## Introduction

Canine impaction can be deﬁned as an infra-osseous position of the canine after the expected eruption time (1,2). The prevalence of maxillary canine impaction ranges from 1% to 5%.6 Impacted canine in the palatal position occurs 3 to 6 times more often than the buccal position. Impacted canines are twice as common in women as in men, and the incidence in the maxilla is more than double compared to the lower jaw (3,4).

Disturbance in the dental lamina, precocious development of the canine in the maxilla and microform of the cleft lip and palate may lead to impaction of maxillary canines (4). Palatally displaced canines have been found to have an autosomal dominant trait with low penetrance and variable expressivity. Palatal canine impactions are related to excessive space in the dental arch, whereas buccal canine impactions have insufficient space to erupt in the dental arch (5). Although there is no consensus about the exact etiology of palatally impacted canines, it appears that the adjacent lateral incisor demonstrates an important role, either because its eruption and dimensions are controlled by the same genes that control the eruption of the canine (genetic theory) or because its position in the arch influences the eruption path of the canine (guidance theory) (6) 

Becker *et al*. (7) related palatal canines to congenitally missing lateral incisors, late formation of the dentition, small lateral incisors, peg-shaped laterals, and short rooted laterals and reported a highly significant relationship between anomalous or absent lateral incisors and palatally displaced canines. In another study, palatally displaced canines were reported to be associated with other impacted and missing teeth, and deep bite with retroclined maxillary incisors. Becker *et al*. found that approximately half of their subjects with palatally displaced canines had delayed dental development (7,8).

Eruption is a physiologic process that influences the normal development of alveolar bone, whereas impaction (impacted tooth) may hinder the regional development of alveolar bone (9,10). Impaction can lead to reduced bone dimensions, or affect dental angulations of the nearby teeth. A few studies (6,10) have compared the impacted area with the contra-lateral area that had adequate canine eruption in the same individual. Kanavakis *et al*. (6) concluded that the root of lateral incisors adjacent to palatal impacted canines is angulated more mesially compared to that of lateral incisors adjacent to normally erupted canines.

With the advent of cone-beam computed tomography (CBCT), more specifically, by rendering three-dimensional (3D) views of teeth and bone at high resolution, detailed characteristic of alveolar bone dimensions can be obtained at the impacted side (10). Finding specific morphological differences in patients with palatally displaced canines could help to revise and maybe add to the accepted interceptive measures: extraction of the deciduous canine (11).

The aim of this study was to compare skeletal and dentoalveolar dimensions in subjects with maxillary unilateral impacted palatal canines versus the unaffected contralateral side using CBCT.

## Material and Methods

This retrospective cross-sectional split mouth design study was approved by the Ethics and Research Committee of the Institution. The sample consisted of full maxillary CBCT scans of 60 patients with unilateral maxillary impacted palatal canines (full FOV of 10 x 10 cm) sourced from the archives of an imaging centre specialising in CBCT imaging. The minimum sample size required was 20 impacted canines per group, determined by a formula to compare 2 means, with a 95% conﬁdence level and 80% test power, when the average difference of RR between groups was 0.5 mm (data from a previous pilot test), and with a standard deviation of 0.64 mm. The inclusion criteria for selection were CBCTs of subjects over 15 years old of both sexes, with canines fully calcified, with a unilateral maxillary canine impaction, complete eruption of the contra-lateral canine and no prior orthodontic treatment. We included impacted canines located in all sectors.17 Exclusion criteria were subjects with previous orthodontic treatment, dento-maxillary traumas, maxillary canine transpositions, agenesis, craniofacial malformations, odontogenic pathologies, and CBCTs of bilateral impacted and bucally impacted canines.

All the CBCT images were taken with the NewTom 3G (QR SRL, Giona,Vila Silverstrini, Verona, Italy) device (90 kV, 10 mAs, 36 sec exp, voxel size 180 µ) with the patient in maximum intercuspation and Frankfort horizontal plane parallel to the floor following common CBCT imaging protocols. All the data sets were exported as Digital Imaging and Communications in Medicine (DICOM) images. Then these data sets were incorporated to the interactive CBCT analysis software. CS 3D Imaging v 3.5.7(Carestream Health) software was used for measurement on Dell Precision T1700 workstation with Dell P2214H monitor with a resolution 1920 X 1080 @ 60 Hz in a dimly lit room. All the measurements were performed on the multi-planar reconstruction (MPR) mode of the CBCT software. The parameters measured are defined in Table 1.

**Table 1:**
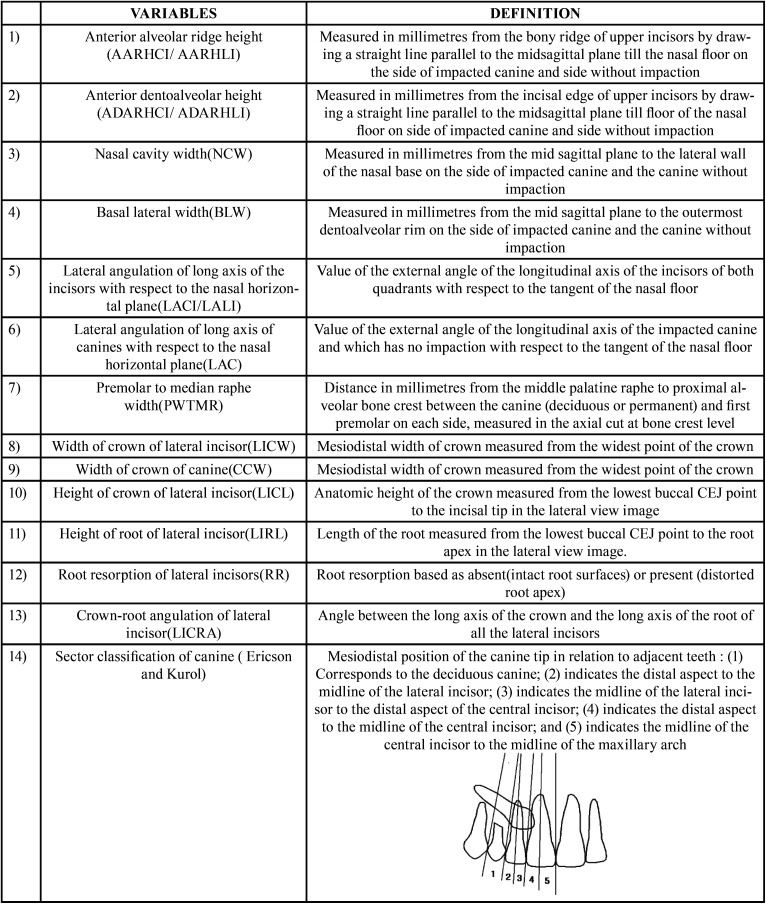
Variables measured on CBCT images.

All CBCT measurements were done by two people, an Oral and Maxillofacial radiologist and one postgraduate student who were trained for the procedure before interpretation. Ten scans per day were evaluated and all the scans were evaluated twice at an interval of ten days.

**Figure 1:**
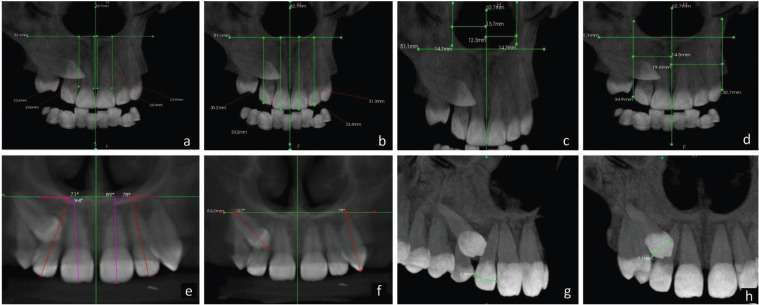
a) Anterior alveolar ridge height measurement. b) Dentoalveolar ridge height measurement. c) Nasal Cavity width measuremet. d) Basal lateral width measurement. e) Lateral angulation of Incisor measurement. f) Lateral angulation of long axis Canine measurement. g) Width of lateral incisor in coronal section. h) Width of canine in coronal section.

**Figure 2:**
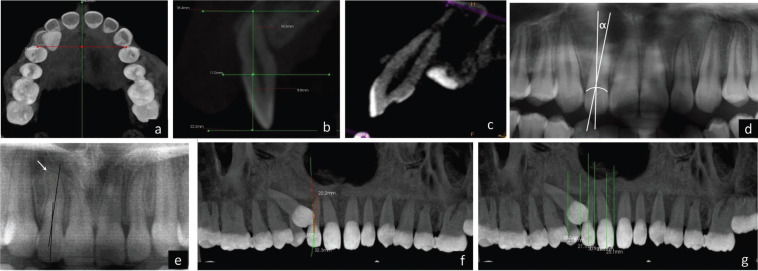
a) Measurement of Premolar to median raphe width. b) Crown and Root Height evaluation on sagittal slice. c) Assessing root resorption of lateral incisor adjacent to canine. d) Angle α used to measure mesio-distal crown root angulation of the lateral incisor. e) Dilaceration of root apex (not regarded when defining the long axis of the root). f) Medially angulated lateral incisor with α > 0. g) Evaluation of Sectors classification in panoramic image reconstructed from axial view of CBCT images.

On the anteroposterior projections derived from the CBCT volume in maximum intensity projections (MIP) in coronal view, parameters (Table 1) were measured keeping a uniform standardized base thickness of 22.1 mm. In the sagittal section views, keeping a uniform standardized base thickness of 180 µ, three horizontal lines were projected, two tangents passing through the apex of the root tip and the crown tip and the third line passing through the buccal CEJ point towards the palatal part of the tooth dividing it into two halves. Length of the crown and root of the lateral incisor (Fig. 1) were measured. Resorption of the lateral incisor caused by the impacted canine was appraised on the volumetric images in multiplanar views on sagittal slice after triangulation of the lateral incisor along the long axis (Fig. 2). Root resorption was evaluated based on the presence of intact (Grade 0) or distorted root surfaces (Grade 1). In the curved slicing mode on the axial image, a manual arc was drawn following the midpoints of the teeth, at a level where both buccopalatal cortices were evident and a reconstructed panaromic view was obtained. Crown-root angulation of the lateral incisor and sector classification of the canine according to Ericson and Kurol were evaluated. When defining the long axis of the lateral incisor root, dilaceration at the root apex was not considered so as to obtain a better representation of the direction of the long axis (Figs. 1,2).

The data was computed and analysed statistically to determine differences between the impacted and non-impacted sides.

-Statistical analysis

The data was subjected to statistical analysis using SPSS version 20.0. The descriptive statistics including the mean, standard deviation, minimum and maximum values were calculated for each of the variables in both the groups. The Shapiro-Wilk test was assessed to determine whether the data had normal distribution. As the data had normal distribution, means were compared using students t test. The significance was set at *p*<0.05. The root resorption in lateral incisor was compared using chi square Test. Reliability between repeat observations was tested by Cronbach’s Alpha and found to be 93.8% which indicated a strong agreement.

## Results

Table 2 shows the descriptive statistics and comparison for all the variables measured at the impacted / non impacted sides. There was no statistical difference between the variables ADARHCI, ADARHLI, AARHCI, AARHLI, LACI, LALI, LICL, LIRL, LICW, CCW and LICRA between the impacted and non-impacted sides. LAC, NCW, BLW and PWTMR variables were found to be significantly different (Table 2).

**Table 2:**
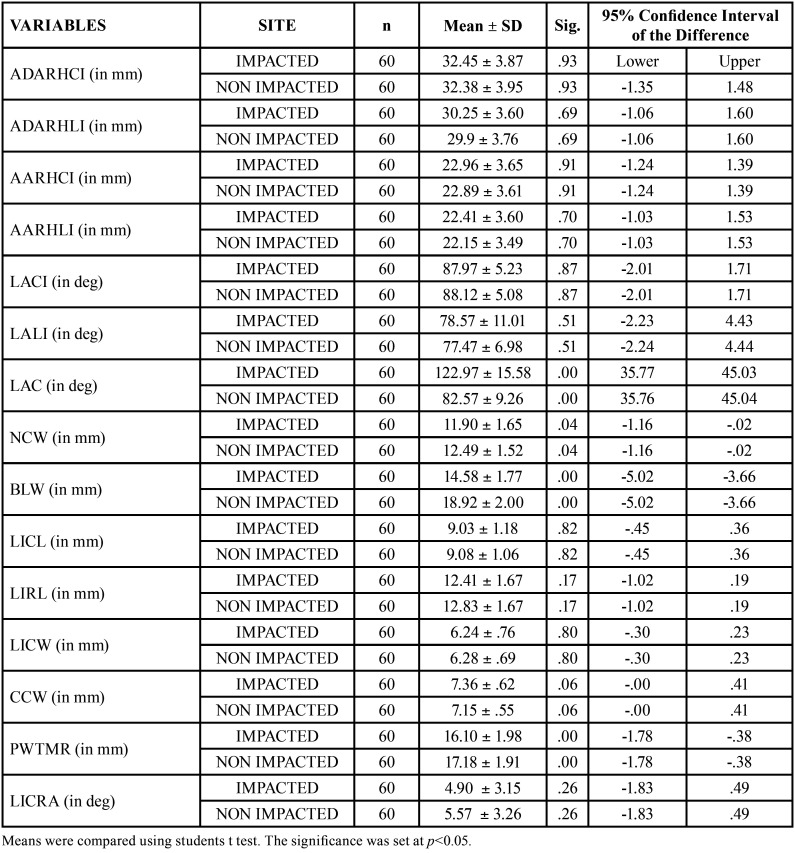
Comparison of variables between impacted and non-impacted side by T-test.

Chi-Square Test was done to evaluate frequencies of root resorption of lateral incisor on impacted side. The difference was insignificant (Table 3).

**Table 3:**
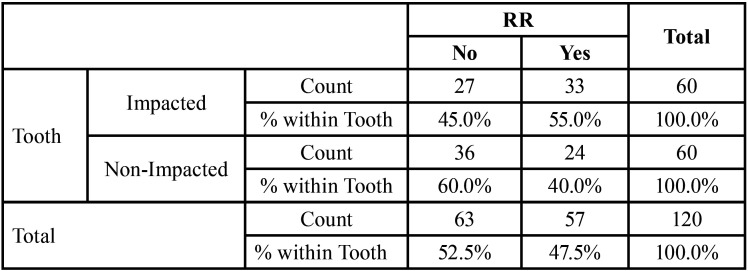
Descriptive statistics to show frequencies of root resorption of lateral incisor on impacted and non-impacted sides.

Chi-Square Test was done to evaluate frequencies of root resorption of lateral incisor on both the sides. The difference was insignificant. 55% cases showed root resorption on impacted side.

The palatal impacted canines were classified into five sectors according to the method of Erricson and Kurol as stated in Table 1 (Table 4).

**Table 4:**
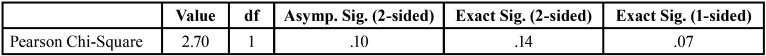
To compare the test values – Chi square test.

Out of 60 impacted canines, the maximum number fell in sector 4 (n = 23, 38.3%), followed by sector 3 (n = 13,21.6%), sector 5 (n = 11,18.3%) and sector 2 (n = 7, 11.6%). Sector 1 showed the least frequency (n = 6,10%).

## Discussion

Early diagnosis of ectopic erupting permanent canines could lead to early interceptive treatment with the goal of preventing impaction and reducing the need for later costly surgical exposure and subsequent orthodontic treatment (11). The relationship between maxillary canine impaction and the morphologic characteristics of the palate was examined in this study. Skeletal and dentoalveolar dimensions in subjects with unilateral maxillary palatal impacted canines were compared between the impacted and non-impacted sides.

On comparing anterior alveolar ridge height and anterior dentoalveolar height of central and lateral incisor between both the sides, the present study showed an insignificant difference. The study by Oleo-Aracena *et al*. (4) showed the same results. They reasoned that the incisor heights should not be affected because the sequence of eruption of incisors is prior to canines. Although their study had subjects of Latin American origin their findings were similar to our study which had subjects of Indian origin.

Our study showed significant differences in nasal cavity width and basal lateral width values, with reduced dimensions on impacted sides as compared to non-impacted sides. This result was in contrast with the findings of Oleo-Aracena *et al*. (4) and Miresmaeili *et al*. (12) who found no difference in these variables between the impacted and non-impacted cases. Saiar *et al*. (13) also found no relationship between the nasal width and PDC. Sar *et al*. (14) found significant differences were observed in the canine angulation, premolar width and basal lateral width between the impacted vs. contra-lateral sides. The disparity in results may have been due to the difference in the sample of the studies; our study and that of Oleo-Aracena *et al*. (4) took cases of unilateral impaction and compared impacted vs non-impacted sides whereas Miresmaeili *et al*. (12) included cases with bilateral and unilateral impacted canines along with a control group. The method of measurement also differed; Oleo-Aracena *et al*. measured nasal cavity width on each side from the anterior nasal spine whereas in this study, the measurement was made from the mid-sagittal plane (4).

Significant differences were observed in premolar to median raphe widths on comparing both the sides, measured as the distance from the mid-palatine raphe to the first premolar. The premolar width on the affected side was significantly lower than on the non-impacted side. This was similar to results of study by Oleo-Aracena *et al*. (4) As they explained, this was because the side of the impacted canine had not been sufficiently developed, compared with the unaffected side where canine erupted normally. Naoumova *et al*. (11) found significantly smaller arch width in the canine region in both unilateral and bilateral canine impaction cases compared with a control group. McConnell *et al*. (15) used dental casts to measure maxillary widths and concluded that patients with PDC have transverse deficiencies.

Narrower arches on the impacted side have been reported by many authors (McConnell *et al*. (15) and Kim *et al*. (16)) whereas some others have reported no difference in arch width (Langberg *et al*. (17), Saiar *et al*. (13) and Anic-Milosevic *et al*. (18)). The reason for these disparities may be due to different methods of measurement; Naoumova *et al*. (11) and Mucedero *et al*. (19) used 3D scans of models whereas this study and some other recent studies (Miresmaeili *et al*. (12), Tadinada *et al*. (10), Oleo-Aracena *et al*. (4), Hong *et al*. (20)) have preferred to measure on CBCT scans to enhance accuracy of measurements in three dimensions. The measurements of arch width have also been made at different levels; Naoumova *et al*. (11), Al-Khateeb *et al*. (21) and Mc-Connell *et al*. (15) measured inter-canine width, Naoumova *et al*. (11) and Al Khateeb *et al*. (21) also measured inter-premolar width, Miresmaeili *et al*. (12), Al Khateeb *et al*. (21) and Kim *et al*. (22) measured inter-molar width whereas Tadinada *et al*. (10) measured bucco-palatal width at 2, 6 and 10 mm. They found that BP width at 2mm above the alveolar crest was significantly less on the side where the canine was impacted but there was no difference at 6 and 10 mm due to presence of the impacted canine (19).

McConnell *et al*. (15) advocated expansion therapy in patients with PDC. However, Naoumaova *et al*. (11) suggested that the absence of atleast one permanent canine in the dental arch is probably the cause of the narrow arch width found in the canine region rather than the narrow arch width being the cause of impaction. Therefore, they did not recommend expansion therapy based solely on the decreased inter-canine or inter-premolar widths. The presence/ absence of the deciduous canine may also affect the arch width measurements. Hence it may be recommended that the clinician should correct the transverse discrepancy where present (11).

In this study, no difference was found in the lateral angulations of the long axis of incisors between the impacted and non-impacted sides. In contrast, statistically significant differences were observed by Oleo-Aracena *et al*. (4) and Liuk *et al*. (23). Oleo-Aracena *et al*. (4) found that the lateral angulation of the long axis of the incisors was lower on the impacted side, presenting disto-angulated incisors on the side of impacted canine and mesio-angulated incisors on the non-impacted side. The variation in the angulation of the incisors may have been due to difference in the vertical level as well as the horizontal overlap (sector) of the impacted canines in the sample of the other studies compared to this study.

In our study the lateral angulation of the long axis of the canines showed significant difference on comparison between impacted and non-impacted side. The impacted side showed a greater angulation of the canine with mesial tipping compared to the non-impacted side. This was similar to results presented by Oleo-Aracena *et al*. (4) and Hanke *et al*. (24).

On assessing the crown-root angulation of lateral incisors, no statistical difference was seen. This result was not in accordance with the study by Kanavakis *et al*. (6) where the long axis of the root of the lateral incisors adjacent to palatal impacted canines formed a more mesial angle to the crown (approximately 2.5°) compared to the lateral incisors adjacent to normally erupted canines.

In the present study, we found no significant differences in the shape of the crown and root of the maxillary lateral incisors between the two sides.

Previous studies have reported significant associations between the morphology of lateral incisors and the presence of a palatally impacted canine. Some tend to support that an abnormally shaped, peg, or missing lateral incisor will cause the adjacent canine to impact by not guiding it into the correct position in the arch (3,16). On the other hand, there are numerous studies (3,4) suggesting that abnormally shaped lateral incisors and palatally impacted canines are both phenotypic expressions of specific genes and therefore tend to occur concomitantly. Results from the present investigation could not be potentially used to support either of the two prevailing theories.

The width of the crown of the maxillary canine was slightly greater on the impacted side but the difference was not statistically significant. This finding was in contrast to the results of the study done by Kim *et al*. (22) who found that the size of the maxillary canine was greater on the impacted side. Their results suggest that there is a possibility that normal eruption might be impaired due to insufficient space in patients with greater crowns of the maxillary canine. According to Jacoby (3) lack of space is associated with buccal impaction of canine whereas there is ‘excessive space in the maxillary arch’ in case of palatally impacted canines. As stated previously Kim *et al*. (22) included both buccal and palatal impacted canines in their sample unlike the present study.

In our study, 55% of the adjacent lateral incisors showed root resorption adjacent to the impacted canines compared to 40% on the non-impacted side. The difference in root resorption incidence was not statistically different. Other studies (6-8) have shown root resorption to vary from 38% to 67%. Our study supports previous studies showing that ectopic eruption of the impacted canine may cause root resorption of the maxillary incisors, most commonly the lateral incisors. Although CBCT allows visualization of the roots in all projections and is presumed to yield a more accurate assessment, it is possible that we have underestimated root resorption because the large FOV diminishes the resolution of the image (pixel size 0.377 in a 12-inch scan vs 0.292 in a 9-inch scan), as stated by Oberoi *et al*. (7). Thus in our study, the lack of significant difference of root resorption of lateral incisors between the impacted and non-impacted sides was probably because of the low resolution of the images, which did not allow for clear depiction of resorption craters.

In the present study, the palatal impacted canines were classified into five sectors according to the method of Erricson and Kurol (25,26) as stated in Table 1. Out of the 60 palatal impacted canines, the maximum number fell in sector 4(n = 23), followed by sector 3 (n = 13), sector 5(n = 11) and sector 2 (n = 7). Sector 1(n = 6) showed the least frequency.

The study by Oleo-Aracena *et al*. (4) included only impacted canines located in sectors 2 and 3; they did not include Sector 1 because according to them, this condition was less frequent than the other two. Due to the unequal distribution of the canines, they could not be evaluated according to sectors.

The limitation of this study was that the palatally displaced canine group from the radiology practice cannot represent the general population. There is a tendency for clinicians to refer only patients with more severely impacted canines or more complicated situations to a radiology practice for further investigation with CBCT. This makes subject selection somewhat biased and any similarity with the general population should be made with caution.

Also, in this study, the most prevalent gender was females confirming that the impacted upper canines are produced twice as common in women than in men. Oleo-Aracena *et al*. (4) found a similar ratio of 2 or 3 to 1, with women being more prevalent in this sample added to etiological factor, is probably the mere fact that women are esthetically more oriented to get orthodontic treatment.

## Conclusions

1. There was a significant difference in some skeletal and dento-alveolar dimensions of the maxillary arch between the impacted and non-impacted sides in cases of unilateral palatal canine impaction.

2. The canines on the impacted side were more mesially angulated compared to the non-impacted side.

3. The nasal cavity width, basal lateral width and premolar to median raphe width were significantly less on the impacted side compared to the non- impacted side.

4. There was no significant difference in the anterior alveolar ridge height of the central incisor, anterior alveolar ridge height of the lateral incisor, anterior dentoalveolar height of the central incisor, anterior dentoalveolar height of the lateral incisor, lateral angulation of long axis of the central incisors , lateral angulation of long axis of the lateral incisors, width of crown of lateral incisor , width of crown of canine, height of crown of lateral incisor, height of root of lateral incisor, root resorption of lateral incisor and the crown-root angulation of lateral incisor between the two groups.
